# Safe transection of aberrant arteries associated with pulmonary sequestrations

**DOI:** 10.1186/s12893-015-0009-1

**Published:** 2015-03-18

**Authors:** Junichi Okamoto, Hirotoshi Kubokura, Jitsuo Usuda

**Affiliations:** Department of Thoracic Surgery, Nippon Medical School Musashikosugi Hospital, 1-396 Kosugi-cho, Nakahara-ku, Kawasaki, 211-8533 Kanagawa Japan; Department of Thoracic Surgery, Nippon Medical School, Tokyo, Japan

**Keywords:** Lobectomy (pulmonary sequestration), Thoracoscopy/VATS, Aberrant-artery handling

## Abstract

**Background:**

Video-assisted thoracoscopic surgery (VATS) lobectomy is increasingly used for pulmonary sequestration; however, there are few descriptions of safe handling of the aberrant artery. Here we clarify the safe handling of an aberrant artery using a clinical review and an experimental model.

**Methods:**

We retrospectively reviewed the records of patients who underwent lobectomy for pulmonary sequestration with aberrant arteries at the Nippon Medical School between January 2008 and December 2010. This was supplemented by an experimental pressure test using vessels obtained from pigs.

**Results:**

We identified four patients with aberrant arteries that were successfully occluded via either stapling. In the experimental model, we divided pig vessels into small-diameter (S) and large-diameter (L) groups. The 1.0-mm-high staples were stronger in the S group than in the L group (*p* = 0.028). In the L group, the 2.0-mm-high staples were stronger than the 1.0-mm staples (*p* = 0.015). Leakage from the staple line was associated with a poorer B-shape of inserted staples.

**Conclusions:**

The techniques described in this report are useful in successful minimally invasive transection of an aberrant artery (other than very thin vessels) when resecting a pulmonary sequestration by stapler only. A detailed investigation should be performed to determine the most appropriate stapler or cartridge.

## Background

Pulmonary sequestration is an uncommon condition characterized by nonfunctioning and abnormal pulmonary parenchyma that has no tracheobronchial airway connection and receives its blood supply from a systemic artery [1]. Definitive treatment is surgical excision, which traditionally involves lobectomy followed by division of the anomalous artery via standard thoracotomy.

Video-assisted thoracoscopic surgery (VATS) lobectomy is increasingly accepted as standard practice. The General Thoracic Surgery Database of the Society of Thoracic Surgeons (STS) showed that by 2006, 32% of lobectomies for primary lung cancer were performed thoracoscopically [2]. This increased experience with VATS has encouraged more complex procedures to be performed using this approach, including segmentectomy, pneumonectomy, lung resection after induction therapy, sleeve resections, and *en bloc* chest wall resection [2].

Surgery is the preferred treatment for pulmonary sequestration; generally via a posterolateral thoracotomy or VATS. However, VATS lobectomy or pulmonary sequestration segmentectomy has been described only in case reports [3-5], with few reports describing the procedure for safely handling the aberrant artery. In the present study, we provide information that can guide surgery in patients with pulmonary sequestration. We also use an experimental model to describe the safe handling of an aberrant artery.

## Methods

This study comprised two stages: First, we retrospectively reviewed the treatment of pulmonary sequestrations with aberrant arteries in our unit. Second, we identified the safest procedure for managing the aberrant arteries on the basis of an experimental model using pig vessels.

### Retrospective case review

**Figure 1:**
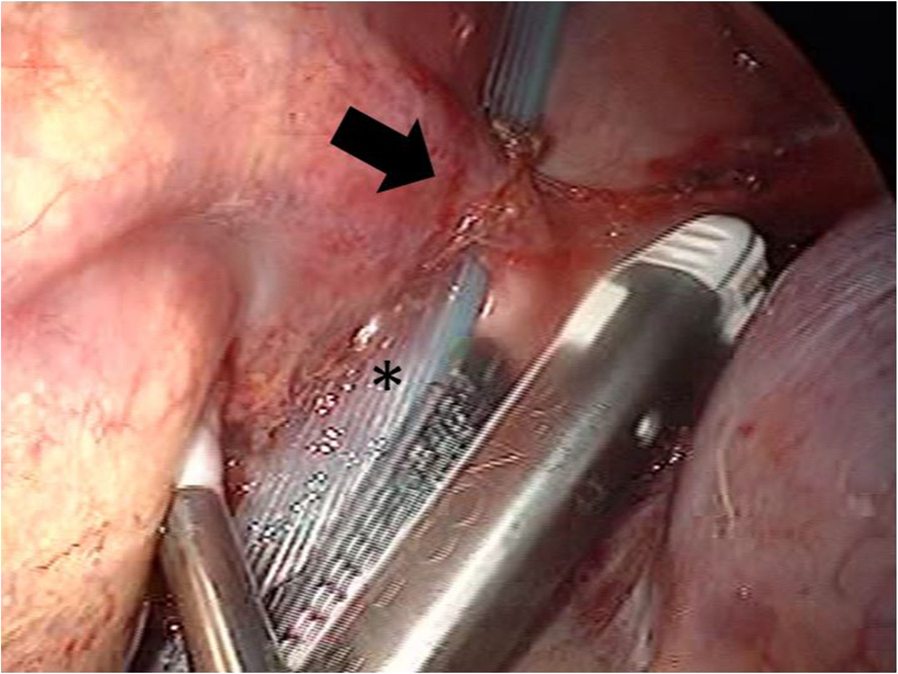
**A skeletonized aberrant artery forming a tunnel between the aberrant artery and the peripheral pleura of the mediastinum.** Arrow showing the aberrant artery and an asterisk (*) showing the Penrose drain placed through the tunnel to ensure safe delivery of the stapler.

We retrospectively reviewed the records of patients who underwent pulmonary lobectomy for pulmonary sequestration at the Nippon Medical School from January 2008 to December 2010. Preoperatively, aberrant arteries were identified using contrast-enhanced computed tomography (CT). All pulmonary lobectomies were performed through VATS, with the latter performed at our institution as previously described [6]. The aberrant arteries were managed with stapling without ligation of the proximal aberrant artery (Table 1). The staplers and staple cartridges were either the Echelon Flex™ 45 or Endo-Cutter ETS 45 (Ethicon Endo-Surgery; Cincinnati, OH, USA) or the Endo-GIA™ Universal (Covidien; Mansfield, MA, USA). Before treating the aberrant artery it was skeletonized to form a tunnel between it and the peripheral pleura of the mediastinum. During VATS, we placed a Penrose drain through the tunnel to ensure safe delivery of the stapler (Figure 1). The operative techniques were standardized for all surgeons. Informed consent for all patients was obtained before the operation, to access patient records. In addition, Nippon Medical School ethics committee permitted all of our college researchers to access and to use the patients’ data for the purpose of each study, after informed consent.

**Table 1:** **Characteristics of patients undergoing surgery for pulmonary sequestration**

**Case No.**	**Side**	**Segment**	**Operation**	**Number of abnormal artery**	**Diameter of abnormal artery**	**Stapler** ^**1**^
**1**	**L**	**S** ^**10**^	**lobectomy**	**1**	**10 mm**	**White**
**2**	**R**	**S** ^**6+10**^	**lobectomy**	**N/A**	**N/A**	**White**
**3**	**L**	**S** ^**10**^	**segmentectomy**	**1**	**20 mm**	**Blue**
**4**	**L**	**S** ^**10**^	**lobectomy**	**1**	**10 mm**	**Gray**

^1^Staple height (closed): white cartridge, 1.0 mm; blue cartridge, 1.5 mm; green cartridge, 0.75 mm.

L: lobectomy; N/A: not available; S: segmentectomy; TS: open thoracotomy; VATS: video-assisted thoracoscopic surgery.

### Experimental model using pig vessels

**Figure 2:**
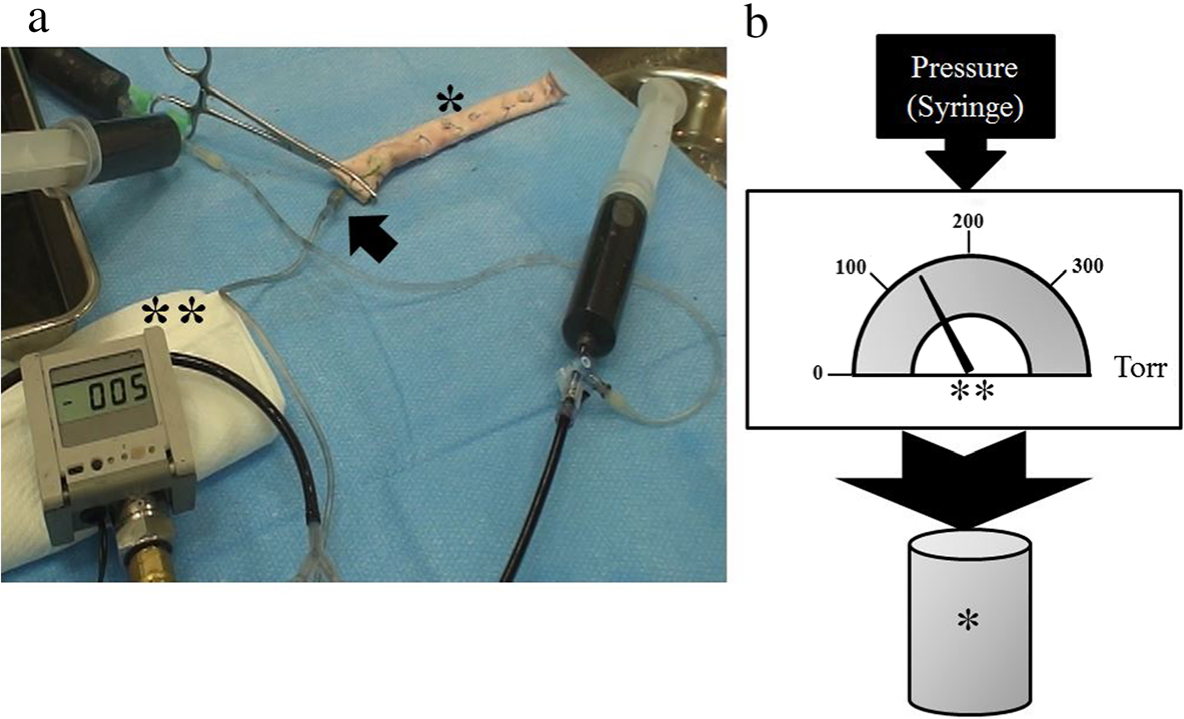
**Pressure experiment using pig arteries. (a)** Pressure-test circuit. **(b)** Experimental schema. Asterisk (*) showing a pig artery: the distal end was closed using a stapler and the proximal end was clamped using Kelly forceps. Arrow showing the pressurized side with a 22-gauge needle in the vessel lumen. Double asterisk (**) showing the measuring instrument (PG208; Nidec Copal Electronics Corp.).

All animal experiments were approved by the Nippon Medical School Animal Research Committee and performed according to the Japanese Guidelines for Animal Experimentation. Pig vessels, seven of which were purchased from a butcher, were used in this study because they have a tissue thickness almost identical to that of the human aberrant artery wall. First, we obtained aortas that were approximately 1–2 cm in diameter and 15 cm long from the specimens. Second, the branches and proximal side of each aorta were ligated to seal against water leakage. Third, we incised and ligated the distal end of the aorta using the Echelon Flex™ 45-mm stapler and three stapler cartridges (Ethicon Endo-Surgery). The staple cartridges were white, blue, and green, and when closed their staples were 1.0 mm, 1.5 mm, and 2.0 mm in height, respectively. The repaired aortas were then divided into small-diameter (S) and large-diameter (L) groups (Table 2). Fourth, an internal-pressure measuring instrument (PG208; Nidec Copal Electronics Corp., Tokyo, Japan) was inserted into the proximal side of the aorta using a 22-gauge needle (Figure 2). Finally, the proximal side was clamped using Kelly forceps to provide further protection against water leakage. The water pressure was increased after sealing using one of the three stapler types on the distal end of the aorta. The pressure at which either the staple line ruptured or water leakage developed was recorded. The experiments were repeated three times for each size of aorta and for each procedure unless the staple line ruptured. A B-shaped staple line, which has previously been described [7], was observed when a water leak from the staple line occurred. Additionally, we checked the pressure twice for simple ligation of an aorta with a diameter of 16.3 mm and a thickness of 0.91 mm. The mean pressures in the S and L groups were compared using a two-tailed paired *t*-test. A *P*-value of < 0.05 was considered significant.

**Table 2:** **Pressure tests of the descending aortas of pigs by staple-cartridge type**

**Group**		**Diameter (mm)**	**Thickness (mm)**	**Staplar** ^**1**^					
				**White**		**Blue**		**Green**	
				**Pressure (mmHg)**	**B shape** ^**2**^	**Pressure (mmHg)**	**B shape** ^**2**^	**Pressure (mmHg)**	**B shape** ^**2**^
**S**	**①**	**13.1**	**1.10**	**405**	**good**	**418**	**good**	**322**	**good**
				**380**		**rupture**		**leakage**	
				**386**		**―**		**―**	
	**②**	**15.1**	**0.91**	**357**	**good**	**300**	**good**	**350**	**good**
				**rupture**		**rupture**		**360**	
				**―**		**―**		**leakage**	
	**③**	**16.2**	**0.99**	**320**	**poor**	**290**	**good**	**220**	**good**
				**170**		**190**		**168**	
				**leakage**		**rupture**		**―**	
	**average**	**14.8 ± 1.6**	**1.00 ± 0.10**	**336.3 ± 86.5**	**―**	**299.5 ± 93.3**	**―**	**284.0 ± 85.3**	**―**
**L**	**④**	**23.6**	**1.70**	**314**	**poor**	**171**	**good**	**323**	**good**
				**leakage**		**169**		**303**	
				**―**		**rupture**		**272**	
	**⑤**	**21.8**	**1.23**	**230**	**poor**	**186**	**good**	**309**	**good**
				**125**		**rupture**		**300**	
				**leakage**		**―**		**298**	
	**⑥**	**20.3**	**1.70**	**220**	**poor**	**269**	**poor**	**223**	**good**
				**149**		**220**		**276**	
				**leakage**		**leakage**		**263**	
	**average**	**21.9 ± 1.6**	**1.54 ± 0.27**	**207.6 ± 74.6**	**―**	**203.0 ± 42.2**	**―**	**285.2 ± 30.2**	**―**

^1^Staple height (closed): white cartridge, 1.0 mm; blue cartridge, 1.5 mm; green cartridge, 2.0 mm.

^2^ *B-shape* refers to the shape of the staple once in situ (i.e., the stapled condition).

## Results

### Results of the retrospective case review

We identified four patients who had been diagnosed after presenting with clinical symptoms such as cough, fever, and hemoptysis caused by pneumonia. Operative details are presented in Table 1. Aberrant arteries were successfully occluded with a stapler in four patients. There were almost no differences between each patient in either blood loss or operative duration (data not shown). Staplers were selected based on the diameter of the aberrant artery. Each aberrant artery was handled with care, and its sheath was removed. Neither procedure caused any postoperative morbidity or mortality.

### Results of the experimental model using pig aortas

Table 2 summarizes the results of the pig experimental model. The S group resisted pressure better than the L group when using the white-cartridge staples (*p* = 0.028). However, there was no significant difference between blue and green cartridges for the S and L groups. In the S group, there was no significant difference among the three cartridge types. In the L group, the green-cartridge staples resisted pressure better than the white-cartridge staples (*p* = 0.015); staple line leakage was caused by poorer B-shapes of those staples than those produced by staples not associated with leakage. In the experiment using ligation only, the two pressures were 303 and 298 mmHg (average, 300.5 mmHg). In the S group, there was no significant difference between ligation and the white, blue, or green cartridge.

## Discussion

Pulmonary sequestration is a relatively uncommon aberration of the lung that is characterized by lung tissue that has a systemic arterial supply [8]. It accounts for up to 6% of congenital pulmonary malformations [8]. If a sequestration is identified, resection is generally required because of the risks of infection and misdiagnosis as a malignant lesion [9]. Intralobar sequestration accounts for 75% of pulmonary sequestrations diagnosed, of which 98% are located in the lower lobes and the majority (58%) on the left side [10]. Thus, accurate location of the aberrant artery is necessary.

To determine the detailed anatomy of the anomalies, we used 3-dimensional (3D) CT imaging (data not shown), which is known to confirm both the presence of the artery and the relevant anatomy [10]. In our cases, we detected the aberrant arteries using 3D CT images to clarify specific anatomical nuances such as the presence of a pleural aberrant artery or a large-diameter artery.

VATS lobectomy has been successfully performed to excise sequestrated lung lobes [8,11-13]. When we first began performing lobectomies for sequestration, we occluded the artery centrally using double ligation before peripherally transecting the artery (data not shown); however, this proved to be unnecessary. We currently use only one stapler intra operatively, even in the presence of very large vessels, and we have not observed stapler failure in any of our VATS lobectomies. Intraoperative bleeding typically occurred during preparation of the aberrant artery and other lobectomy procedures in a previous report [12]. Chung *et al.* reported that when the renal artery and vein were stapled simultaneously using an endovascular GIA stapler (Echelon Flex 60 mm, 2.5 load Endo-cutters; Ethicon Endo-Surgery), there was no clinical evidence of bruit 12 months postoperatively [14]. That is, there was no evidence of stapling-related morbidity from the transection of systemic arteries such as the renal artery.

In fact, in the present study, there were no significant differences between ligation and stapling in terms of morbidity or mortality. Although endoscopic stapling is widely used to transect large-diameter vessels, particular care must be taken when it is used to transect an artery feeding from the aorta. This is to prevent massive bleeding caused by incomplete closure of the artery, which can occur if the endoscopic staple cutter malfunctions. Fortunately, this did not occur in the present cases. However, Liu *et al.* reported that one of their VATS cases was converted to thoracotomy because of injury to the aberrant artery [15]. Additionally, when the aberrant artery was thickened or had become fragile because of recurrent infections, they often ligated the artery proximally using silk sutures before cutting it with a stapler in order to ensure a solid stump [15]. In the case, that the aberrant artery is fragile, it may be necessary to ligate the aberrant artery prior to stapling.

In this experiment, we selected three types of stapling cartridges: white, blue, and green. Although blue and green cartridges are not routinely used, in the experimental portion of the present study, the pig vessels were denatured because of preservation such as freezing. Furthermore, we used pig vessels from the descending aorta, which were of larger diameter and thickness than are aberrant arteries associated with pulmonary sequestrations. We therefore had to use blue and green cartridges in our experiment. The results of our experimental model with pig aortas reveal that it is necessary to select the stapling cartridge based on the size of the aberrant artery (Table 1). In fact, an unsuitable cartridge appeared to weaken the staple line, whereas appropriate selection resulted in a solid stump capable of withstanding pressures of approximately 300 mmHg. In other words, using only adequate stapler cartridge made us handle the water leakage from pig aortas in this experiment. Therefore, it is acceptable that an aberrant artery can be successfully transected with minimal invasion when resecting pulmonary sequestrations with a stapler alone. In fact, we achieve similar results for both ligation and stapling in clinical cases. Final, VATS procedure using a stapler and an adequate cartridge device is increasingly employed.

## Conclusions

The techniques described in this report are suitable for transection of all vessels, except those that are very thin, when the appropriate stapler is selected. Before treatment, 3D CT is necessary to select the optimal stapler and cartridge.

## Abbreviations

CT: Computed tomography
3D: 3-Dimensional
STS: Society of thoracic surgeons
VATS: Video-assisted thoracoscopic surgery
